# Hazardous and Harmful Alcohol Use and Associated Factors in Tuberculosis Public Primary Care Patients in South Africa

**DOI:** 10.3390/ijerph9093245

**Published:** 2012-09-05

**Authors:** Karl Peltzer, Julia Louw, Gugu Mchunu, Pamela Naidoo, Gladys Matseke, Bomkazi Tutshana

**Affiliations:** 1 HIV/STI and TB (HAST) Research Programme, Human Sciences Research Council, Pretoria, Durban and Cape Town 8000, South Africa; Email: jlouw@hsrc.ac.za (J.L.); gmchunu@hsrc.ac.za (G.M.); pnaidoo@hsrc.ac.za (P.N.); gmatseke@hsrc.ac.za (G.M.); btutshana@hsrc.ac.za (B.T.); 2 Department of Psychology, University of Limpopo, Turfloop 06854, South Africa; 3 Department of Psychology, University of the Western Cape, Cape Town 8000, South Africa

**Keywords:** alcohol misuse, tobacco use, associated factors, tuberculosis patients, public primary care, South Africa

## Abstract

The aim of this study was to assess the prevalence of hazardous and harmful alcohol use and associated factors among patients with tuberculosis in South Africa. In a cross-sectional survey new tuberculosis (TB) and TB retreatment patients were consecutively screened using the Alcohol Use Disorder Identification Test (AUDIT) within one month of anti-tuberculosis treatment. The sample included 4,900 (54.5% men and women 45.5%) tuberculosis patients from 42 primary care clinics in three districts. Results indicate that, overall 23.2% of the patients were hazardous or harmful alcohol drinkers, 31.8% of men and 13.0% of women were found to be hazardous drinkers, and 9.3% of men and 3.4% of women meet criteria for probable alcohol dependence (harmful drinking) as defined by the AUDIT. Men had significantly higher AUDIT scores than women. In multivariable analyses it was found that among men poor perceived health status, tobacco use, psychological distress, being a TB retreatment patient and not being on antiretroviral therapy (ART), and among women lower education, tobacco use and being a TB retreatment patient were associated with hazardous or harmful alcohol use. The study found a high prevalence of hazardous or harmful alcohol use among tuberculosis primary care patients. This calls for screening and brief intervention and a comprehensive alcohol treatment programme as a key component of TB management in South Africa.

## 1. Introduction

South Africa has 0.7% of the World’s population and 28% of the World’s population of HIV and TB co-infected individuals. It has been estimated that approximately 60% of people with TB are co-infected with HIV [1]. Co-infected patients have almost double the chances of getting multidrug-resistant TB (MDR-TB) as well as extensively drug-resistant TB (XDR-TB). These patients also have a high mortality rate due to co-infection with HIV [2]. In addition to alcohol’s role in the onset of TB, there is also strong evidence of a negative influence of heavy drinking/alcohol use disorder (AUD) on the clinical course of TB, higher relapse rates and experiencing the most destructive forms of TB [3,4]. 

Prevalence of alcohol use disorders among TB patients have ranged from 10% to 50% in studies carried out in Australia, Canada, Russia, Switzerland, and the USA [4]. There is only a few studies which have assessed alcohol use disorders in tuberculosis patients in low and middle income countries: Kazakhstan: 4% alcohol abusers [5]; Russia: 24–62% alcohol abuse/dependent [6,7,8,9,10]; India: 14.9–32% alcohol abusers/alcoholics [11,12,13]; Brazil: 14–24% alcohol abusers [14,15] and South Africa: 31–62% alcohol misuse [16]. Alcohol use was estimated to have been responsible for 939,000 disability-adjusted life-years lost in South Africa for TB and HIV/AIDS alone in 2004 (253,000 for women, 687,000 for men). This figure corresponds to 4.6% of the overall disease burden in South Africa (2.5% for women, 6.6% for men) [17]. Factors associated with alcohol use in tuberculosis patients include: (male) gender [10,13], older age [13], being married [13], school education [13], middle income [13], treatment category [13] and TB medication non-adherence [5,9,10,11,18,19]. Smoking plus alcohol abuse was found as a probable risk factor for pulmonary tuberculosis in Chengdu, China [20]. Previous studies also found that anxiety or depression and tobacco use were associated with alcohol use disorders in general patients [21,22,23,24].

There is a lack of information on prevalence of alcohol use and AUD amongst TB patients and its impact on adherence and disease progression, in particular in low and middle income countries [3,4]. Detecting alcohol use disorders, specifically alcohol abuse and dependence, provides a critical opportunity for early intervention efforts to reduce adverse impacts of consumption. It is against this background that the reported study was carried out. The findings from this study will assist the national TB programme in South Africa to develop effective intervention strategies for TB patients with problems related to alcohol use. The aim of the study is to estimate prevalence of recent alcohol use and hazardous or harmful drinking among TB patients attending public primary care clinics in South Africa.

## 2. Methodology

### 2.1. Sample and Procedure

This is a cross-sectional survey with tuberculosis patients in public primary care clinics in South Africa. Three provinces, in South Africa, with the highest TB caseload were selected for inclusion in the study. One district in each of three provinces with the highest TB caseloads were ultimately included. These districts were Siyanda in Northern Cape Province, Nelson Mandela. Metro in the Eastern Cape Province, and EThekwini in KwaZulu-Natal Province. Within each of these three study districts 14 primary health care facilities were selected on the basis of the highest TB caseloads per clinic, in all 42 clinics. The type of health facilities were public primary health care clinic or community health centre. 

All new TB and retreatment patients were consecutively screened using the Alcohol Use Disorder Identification Test (AUDIT) within one month of anti-tuberculosis treatment. The screening interview was conducted by trained external research assistants for a period of 6 months in all 42 clinics in 2011. A health care provider who identified a new TB treatment or retreatment patient (within one month on treatment) and 18 years and above informed the patient about the study and referred the patient for participation if interested. A research assistant asked for permission/consent from patients attending the primary care facility to participate in the screening interview. Because of the stigma associated with alcohol consumption, individuals may feel defensive when responding to questions about their drinking and answer inaccurately. 

To increase the reliability of the AUDIT, researchers have suggested putting alcohol consumption in the context of other health-related behaviours [25]. Therefore, the interviewer administered questionnaire included questions on mental and physical health status, tobacco use and chronic diseases. We have received ethical approval from the Human Sciences Research Council Research Ethics Committee (Protocol REC No.1/16/02/11). The Department of Health in South Africa has also provided approval for this study.

### 2.2. Measures

#### 2.2.1. Socioeconomic Characteristics

A researcher-designed questionnaire is used to record information on participants’ age, gender, educational level, marital status, income, employment status, dwelling characteristics and residential status. *Poverty* was assessed with five items on the availability or non-availability of shelter, fuel or electricity, clean water, food and cash income in the past week. Response options ranged from 1 = “Not one day” to 4 = “Every day of the week”. Poverty was defined as higher scores on non-availability of essential items. The total score ranged from 5 to 20, 5 = being low, 6−12 = medium and 13−20 = high poverty. Cronbach alpha for this poverty index was 0.89 in this sample.

#### 2.2.2. Alcohol Consumption

The 10-item Alcohol Disorder Identification Test (AUDIT) [26] assesses alcohol consumption level (three items), symptoms of alcohol dependence (three items), and problems associated with alcohol use (four items). Heavy episodic drinking is defined as the consumption of six standard drinks (10 g of alcohol) or more on a single occasion [26]. In South Africa a standard drink is 12 g of alcohol. Because AUDIT is reported to be less sensitive at identifying risk drinking in women [27], the cut-off points of binge drinking for women (four units) were reduced by one unit as compared with men (five units), as recommended by Freeborn *et al*. [27]. 

Responses to items on the AUDIT are rated on a 4-point Likert scale from 0 to 4, for a maximum score of 40 points. Higher AUDIT scores indicate more severe levels of risk; a score of 8 indicates a tendency to problematic drinking or hazardous or harmful drinking. The AUDIT was developed by the World Health Organization as an effective screening instrument for alcohol use problems among patients seeking primary care for other medical problems in international settings including African countries (Kenya and Zimbabwe) [25,26] and has been validated in HIV patients in South Africa showing excellent sensitivity and specificity in detecting MINI-defined dependence/abuse (area under the receiver-operating characteristic curve, 0.96) [28] and among TB and HIV patients in primary care in Zambia demonstrating good discriminatory ability in detecting MINI-defined current AUDs (AUDIT = 0.98 for women and 0.75 for men) [29]. Cronbach alpha for the AUDIT in this sample was 0.92, indicating excellent reliability. Hazardous drinking is defined as a quantity or pattern of alcohol consumption that places patients at risk for adverse health events, while harmful drinking is defined as alcohol consumption that results in adverse events (e.g., physical or psychological harm) [30].

#### 2.2.3. Tobacco Use

Two questions were asked about the use of tobacco products: (1) Do you currently use one or more of the following tobacco products (cigarettes, snuff, chewing tobacco, cigars, *etc*.)? Response options were “yes” or “no”; (2) In the past month, how often have you used one or more of the following tobacco products (cigarettes, snuff, chewing tobacco, cigars, *etc*.)? Response options were once or twice, weekly, almost daily and daily. 

#### 2.2.4. Kessler Psychological Distress Scale (K-10)

The Kessler Psychological Distress Scale (K-10) was used to measure global psychological distress, including significant pathology which does not meet formal criteria for a psychiatric illness [31,32]. This scale measures the following symptoms over the preceding 30 days by asking: “In the past 30 days, how often did you feel: nervous; so nervous that nothing could calm you down; hopeless; restless or fidgety; so restless that you could not sit still; depressed; that everything was an effort; so sad that nothing could cheer you up; worthless; tired out for no good reason?” The frequency with which each of these items was experienced was recorded using a five-point Likert scale ranging from “none of the time” to “all the time”. 

The total score of the scale is summed with higher scores reflecting a greater degree of psychological distress, range 10–40. The K-10 has been shown to capture variability related to non-specific depression, anxiety and substance abuse, but does not measure suicidality or psychoses [33]. This scale serves to identify individuals who are likely to meet formal definitions for anxiety and/or depressive disorders, as well as to identify individuals with sub-clinical illness who may not meet formal definitions for a specific disorder [31]. 

This scale is increasingly used in population mental health research and has been validated in multiple settings [34] including in a population-based survey in South Africa [35]. The K10 demonstrated moderate discriminating ability in detecting depression and anxiety disorders in the general population in South Africa; evidenced by area under the receiver operating curves of 0.73 and 0.72 respectively, with a cut off of 16 [35]. We examined the K-10 scale using the cut off of 16 indicating psychological distress. The internal reliability coefficient for the K-10 in this study was alpha = 0.92.

#### 2.2.5. Health Status. Perceived General Health

Participants were asked, “In general, would you say your health is: excellent, very good, good, fair or poor?” The measure was categorized based on participant response (very good = excellent/very good, good, and poor = fair/poor). TB treatment status, HIV status and antiretroviral treatment was assessed by self-report and from medical information. Patients were also asked about a list of chronic and other illness conditions they had been diagnosed with such as diabetes.

### 2.3. Data Management and Analysis

Data from the questionnaires were entered manually and verified. The verification process included double data entry of all questionnaires and its fields, doing programmed range checks by computer to identify outlying values, checking for missing values, and checking for inconsistencies in the data. Data were analyzed using Statistical Package for the Social Sciences (SPSS) for Windows software application programme version 19.0. Frequencies, means, standard deviations, were calculated to describe the sample. Data were checked for normality distribution and outliers. For non-normal distribution non-parametric tests were used. Associations of hazardous or harmful alcohol use were identified using logistic regression analyses. Following each univariate regression, multivariable regression models were constructed. Independent variables from the univariate analyses were entered into the multivariable model if significant at *P* < 0.05 level. Logistic regression was conducted for men and for women separately. For each model, the R^2^ are presented to describe the amount of variance explained by the multivariable model. Probability below 0.05 was regarded as statistically significant.

## 3. Results and Discussion

### 3.1. Sample Characteristics

From the total sample (N = 4,935) included in the study 35 subjects (0.7%) refused to participate, so the final sample included 4,900, 54.5% men and 45.5% women, with a mean age of 36.2 years (SD = 11.5), range 18 to 93 years. Almost two-thirds of the participants (65.2%) were between 25 to 44 years old, the majority (72.7%) was never married, 27.7% had completed secondary education, and 17% scored high on the poverty index. Regarding the TB treatment status, 76.6% were new TB patients and 23.4% were TB retreatment patients. From those who had tested for HIV, 59.9% were HIV positive, 22% of the HIV positive patients were on antiretroviral therapy, 9.6% had never tested for HIV. One in five patients (20.0%) were daily or almost daily tobacco users, 81.0% indicated having psychological distress, 4.5% had been diagnosed with diabetes and 46.3% perceived their health status as fair or poor (see Table 1).

**Table 1:** Sample characteristics.

Variable	Total sample (n = 4,900)		Hazardous or harmful alcohol use					
			Total		Men (n = 2,671) (54.5%)		Women (n = 2,229) (45.5%)	
	N	%	N	%	N	%	N	%
**Sociodemographics**								
*Age *								
18–34	1,769	36.6	378	21.6	259	31.4	114	12.6
35–44	2,018	41.9	457	23.2	341	30.6	109	13.1
45 or more	1,040	21.5	268	26.2	211	34.9	51	13.0
Missing	73							
*Marital status*								
Never married	3,356	72.7	760	23.0	560	32.9	195	12.4
Married/cohabitating	101	21.5	237	24.1	176	30.0	54	14.2
Separated/divorced/widowed	275	5.8	64	23.5	37	26.4	21	17.2
Missing	268							
*Education*								
Grade 7 or less	1,269	26.3	355	28.4	255	34.7	94	19.0
Grade 8–11	2,213	45.9	543	24.9	299	33.1	135	14.3
Grade 12 or more	1,336	27.7	205	15.5	153	25.2	49	7.1
Missing	82							
*Poverty index (5–20)*								
Low (5)	1,617	35.0	313	19.5	243	27.8	65	9.2
Medium (6–12)	2,227	48.2	512	23.7	384	33.2	129	12.8
High (13–20)	776	16.9	208	27.3	451	35.4	52	15.9
Missing	280							
**Health variables**								
New TB patient	3,707	76.6	777	21.2	566	29.4	194	11.6
TB retreatment patient	1,128	23.4	333	30.0	247	39.1	83	17.9
HIV positive	2,619	59.9	552	21.4	367	30.4	178	13.2
HIV negative	1,759	40.1	434	25.0	392	32.4	81	12.5
Daily or almost daily tobacco use	980	20.0	485	49.9	401	52.0	74	40.0
Psychological distress (Kessler 10 > 15)	3,970	81.0	941	24.2	686	33.0	230	13.6
*Perceived health status *								
Excellent/very good	928	19.1	190	20.7	140	26.9	47	12.3
Good	1,667	34.6	395	23.9	287	33.1	102	13.4
Fair/poor	2,238	46.3	524	23.8	383	32.8	130	13.0
Missing	67							
Diagnosed with diabetes	194	4.5	37	19.2	27	30.7	9	9.1
On antiretroviral therapy	906	22.0	163	18.2	105	26.6	57	11.5

### 3.2. Alcohol Use

Using a cut-off score of 8 to 19 for the AUDIT analysis indicated that 22.5% of all men and 9.5% of all women were classified as hazardous drinkers, and 9.3% of men and 3.4% of women meet criteria for probable alcohol dependence (harmful drinking) (with an AUDIT score of 20 or more) as defined by AUDIT. Overall 23.2% of the patients were hazardous or harmful alcohol users, 31.8% among men and 13.0% among women. Men had significantly higher AUDIT scores than women (see Table 2).

**Table 2:** Alcohol use by sex.

Group	AUDIT score	Total N (%)	Men N (%)	Women N (%)	^χ2 ^or *	*P*
Abstainers and low-risk drinkers	0–7	3,688 (76.8)	1,759 (68.2)	1,878 (81.6)	233.41	0.000
High risk drinkers	8–19	799 (16.6)	579 (22.5)	206 (9.5)	234.10	0.000
Probable alcohol dependence	20+	321 (6.6)	241 (9.3)	74 (3.4)		
Hazardous or harmful drinkers	8+	1,120 (23.2)	820 (31.8)	280 (13.0)	233.41	0.000
		M (SD)	M (SD)	M (SD)		
Total AUDIT score		4.3 (8.1)	5.7 (8.1)	2.4 (6.0)	*	0.000

* Mann-Whitney U test = *P* < 0.001.

### 3.3. Results of Logistic Regression: Associations between Sociodemograhic and Health Variables and Hazardous or Harmful Alcohol Use

Univariate analyses found that among men lower formal education, greater poverty, poorer perceived health status, being a TB retreatment patient, tobacco use and not being on ART were associated with hazardous or harmful alcohol use, while among women lower formal education, greater poverty, being on TB retreatment and tobacco use were associated with hazardous or harmful alcohol use. In multivariable analyses it was found that compared to male participants with excellent or very good health status, male participants with fair or poor health status were 1.34 times more likely to be hazardous or harmul alcohol users. Compared to male participants who were not daily or almost daily tobacco users, male participants who were daily or almost daily tobacco users were 3.71 times more likely to be hazardous or harmul alcohol users. Male participants who had psychological distress were 1.40 more likely to be hazardous or harmful alcohol users compared to male participants who did not have psychological distress. Compared to male participants who were new TB patients, male participants who were TB retreatment patients were 1.30 more likely to be hazardous or harmul alcohol users, and male participants who were on ART were 34% less likely to be hazardous or harmful alcohol users than male participants who were not on ART. Compared to female participants who attained Grade 7 or less education, female participants who had Grade 12 or more education were 67% less likely to be hazardous or harmful alcohol drinkers. Further, compared to female participants who were new TB patients, female participants who were TB retreatment patients were 1.44 more likely to be hazardous or harmul alcohol users, and female participants were daily or almost daily tobacco users were 6.06 times more likely to be hazardous or harmul alcohol users (see Table 3).

**Table 3:** Results of logistic regression: Associations between sociodemograhic and health variables and hazardous or harmful alcohol use.

Factors	Men		Women	
	Cr OR (95% CI)^ a^	Adj OR (95% CI)^ a,b^	Cr OR (95% CI)^ a^	Adj OR (95% CI)^ a,c^
**Sociodemographics**				
Age				
18–30	1.00	1.00	1.00	1.00
31–44	0.96 (0.79–1.17)	0.89 (0.70–1.13)	1.05 (0.79–1.39)	1.01 (0.76–1.34)
45 or more	1.17 (0.94–1.46)	1.03 (0.72–1.43)	1.04 (0.73–1.47)	1.00 (0.70–1.42)
*Marital status*				
Not married	1.00	---	1.00	---
Married/cohabitating	0.88 (0.72–1.08)		1.17 (0.84–1.62)	
Separated/divorced/widowed	0.74 (0.50–1.09)		1.47 (0.89–2.40)	
*Education*				
Grade 7 or less	1.00	1.00	1.00	1.00
Grade 8–11	0.93 (0.77–1.13)	1.34 (0.99–1.83)	0.71 (0.53–0.95) *	0.73 (0.52–1.04)
Grade 12 or more	0.64 (0.50–0.81) ***	1.08 (0.74–1.58)	0.32 (0.22–0.47) ***	0.33 (0.21–0.51) ***
*Poverty *				
Low	1.00	1.00	1.00	1.00
Medium	1.29 (1.06–1.56) **	1.11 (0.88–1.99)	1.45 (1.06–1.98)	1.35 (0.97–1.89)
Poverty high	1.43 (1.11–1.83) **	1.11 (0.81–1.51)	1.85 (1.25–2.73) **	1.38 (0.90–2.10)
**Health variables**				
*Perceived health status *				
Excellent/very good	1.00	1.00	1.00	---
Good/	1.35 (1.06–1.71) *	1.42 (1.06–1.90) *	1.10 (0.76–1.60)	
Fair/poor	1.33 (1.06–1.67) *	1.34 (1.00–1.80) *	1.07 (0.75–1.53)	
New TB patient	1.00	1.00	1.00	1.00
TB retreatment patient	1.54 (1.28–1.86) ***	1.30 (1.03–1.65) *	1.67 (1.26–2.21) ***	1.44 (1.05–1.97) *
Daily or almost daily tobacco use	3.59 (3.01–4.30) ***	3.71 (3.00–4.59) ***	5.72 (4.12–7.94) ***	6.06 (4.02–9.14) ***
Psychological distress (K−10 > 15)	1.35 (1.09–1.68) **	1.40 (1.07–1.84) *	1.36 (0.96–1.92)	---
Diabetes	0.91 (0.57–1.44)	---	0.66 (0.33–1.32)	---
HIV negative	1.00	---	1.00	---
HIV positive	0.91 (0.76–1.09)		1.07 (0.81–1.42)	
On ART	0.77 (0.60–0.98) *	0.76 (0.58–0.98) *	0.91 (0.66–1.25)	---

^a^ Using “enter” logistic regression selection of variables; ^b ^For men Hosmer and Lemeshow Chi-square 9.59, df 8, 0.295; Cox and Snell R^2 ^0.09; Nagelkerke R^2^ 0.13; ^c ^For women Hosmer and Lemeshow Chi-square 5.56, df 8, 0.696; Cox and Snell R^2^ 0.06; Nagelkerke R^2 ^0.11; * *p* < 0.05; ** *p *< 0.01; *** *p *< 0.001.

## 4. Discussion

This study provides new information on the prevalence of hazardous or harmful alcohol use in patients being treated for TB. Our results found high rates of hazardous or harmful drinking (23.2%) among tuberculosis public primary care patients in South Africa, which is consistent with studies conducted in low and middle income countries [6,7,8,9,10,11,12,13,14,15,16]. Previous studies using the same alcohol measure (the AUDIT) found lower rates of hazardous or harmful alcohol use in general public primary care patients in South Africa 13.3% [36] and 19.2% [37] and in a national population-based survey in South Africa (9%) [38]. The higher prevalence of hazardous and harmful alcohol use among tuberculosis than general primary care patients may be in line with the causal link between alcohol abuse and tuberculosis [3,4,39].

Further, this study found, based on multivariable analysis, in concordance with other studies that male gender [13] and tobacco use [20,23,24] were associated with hazardous or harmful alcohol use. In univariate analysis this study found greater poverty, as found in other studies [13], to be associated with alcohol use. There is a concern as alcohol users from poor households spend large proportions of their income on alcohol and tobacco potentially leading to a vicious cycle between treatment outcomes and the patient’s low financial situation [13,40]. Further, the study showed that being on TB retreatment patient was associated with hazardous or harmful alcohol use. Also other studies found TB retreatment patients category [13] and TB medication non-adherence [5,9,10,11,18], which may have led to retreatment, to be associated with hazardous or harmful alcohol use. This seems to confirm evidence of a negative influence of heavy drinking/alcohol use disorder (AUD) on the clinical course of TB, higher relapse rates and experiencing the most destructive forms of TB [3,4]. In agreement with other studies [14], this study found no difference regarding association with HIV and alcohol consumption. In agreement with other studies in general patients [21,22,23,24,41], this study found among men an association between anxiety or depression with alcohol use disorders.

## 5. Study Limitations

Caution should be taken when interpreting the results of this study because of certain limitations. As this is a cross-sectional study, causality between the compared variables cannot be concluded. Further, we relied on self-report data; thus, the alcohol prevalence rates are subject to biases such as problems recalling alcohol use and social desirability of responses [42]. However, the AUDIT has been found to be reliable and acceptable for screening use internationally [43], in South Africa [28] and among tuberculosis patients in Zambia [29], and to have validity similar to other established self-report instruments [44]. Other limitations include that no drug screening and no standardized psychiatric measures (such as the Structured Interview for DSM-IV Axis I disorders) were used.

## 6. Conclusions

The study found a high prevalence of hazardous or harmful alcohol use among tuberculosis primary care patients. The identification of factors such as daily or almost daily tobacco users and being a TB retreatment patient should guide the design and implementation of programmes aimed to reduce hazardous or harmful alcohol use. The study brings about a number of clinical and policy issues. First, TB health facilities need to develop specific programmes to screen for and detect alcohol use disorders in patients being treated for TB. Second, South Africa needs to develop a comprehensive approach to the treatment of alcohol problems in TB patients including brief interventions delivered by nurses or community health workers and medical practitioners may wish to consider using pharmacotherapy for alcohol dependence. Third, programmes need to integrate prevention of HIV infection in this population [6].

## References

[1] World Health Organisation (WHO) (2010). Reference. Global TB Control Report 2010.

[2] Department of Health (2007). Reference. Tuberculosis Strategic Plan for South Africa 2007-2011.

[3] Rehm J., Samokhvalov A.V., Neuman M.G., Room R., Parry C., Lönnroth K., Patra J., Poznyak V., Popova S. (2009). The association between alcohol use, alcohol use disorders and tuberculosis (TB). A systematic review. BMC Public Health.

[4] Lönnroth K., Williams B.G., Stadlin S., Jaramillo E., Dye C. (2008). Alcohol use as a risk factor for tuberculosis—A systematic review. BMC Public Health.

[5] Bumburidi E., Ajeilat S., Dadu A., Aitmagambetova I., Ershova J., Fagan R., Favorov M.O. (2006). Centers for Disease Control and Prevention (CDC): Progress toward tuberculosis control and determinants of treatment outcomes—Kazakhstan, 2000-2002. MMWR Morb. Mortal. Wkly. Rep..

[6] Fleming M.F., Krupitsky E., Tsoy M., Zvartau E., Brazhenko N., Jakubowiak W., McCaul M.E. (2006). Alcohol and drug use disorders, HIV status and drug resistance in a sample of Russian TB patients. Int. J. Tuberc. Lung Dis..

[7] Krupitsky E.M., Zvartau E.E., Lioznov D.A., Tsoy M.V., Egorova V.Y., Belyaeva T.V., Antonova T.V., Brazhenko N.A., Zagdyn Z.M., Verbitskaya E.V. (2006). Co-morbidity of infectious and addictive diseases in St. Petersburg and the Leningrad Region, Russia. Eur. Addict. Res..

[8] Gelmanova I.Y., Keshavjee S., Golubchikova V.T., Berezina V.I., Strelis A.K., Yanova G.V., Atwood S., Murray M. (2007). Barriers to successful tuberculosis treatment in Tomsk, Russian Federation: Non-adherence, default and the acquisition of multidrug resistance. Bull. World Health Organ..

[9] Jakubowiak W.M., Bogorodskaya E.M., Borisov S.E., Danilova I.D., Kourbatova E.V. (2007). Risk factors associated with default among new pulmonary TB patients and social support in six Russian regions. Int. J. Tuberc. Lung Dis..

[10] Shin S.S., Mathew T.A., Yanova G.V., Fitzmaurice G.M., Livchits V., Yanov S.A., Strelis A.K., Mishustin S.P., Bokhan N.A., Lastimoso C.S. (2010). Alcohol consumption among men and women with tuberculosis in Tomsk, Russia. Cent. Eur. J. Public Health.

[11] Santha T., Garg R., Frieden T.R., Chandrasekaran V., Subramani R., Gopi P.G., Selvakumar N., Ganapathy S., Charles N., Rajamma J. (2002). Risk factors associated with default, failure and death among tuberculosis patients treated in a DOTS programme in Tiruvallur District, South India, 2000. Int. J. Tuberc. Lung Dis..

[12] Kolappan C., Gopi P.G., Subramani R., Narayanan P.R. (2007). Selected biological and behavioural risk factors associated with pulmonary tuberculosis. Int. J. Tuberc. Lung Dis..

[13] Suhadev M., Thomas B.E., Raja Sakthivel M., Murugesan P., Chandrasekaran V., Charles N., Durga R., Auxilia M., Mathew T.A., Wares F. (2011). Alcohol Use Disorders (AUD) among tuberculosis patients: A study from Chennai, South India. PLoS One.

[14] Paixão L.M., Gontijo E.D. (2007). Profile of notified tuberculosis cases and factors associated with treatment dropout. Rev. Saude Publica.

[15] Salles C.L., Conde M.B., Hofer C., Cunha A.J., Calçada A.L., Menezes D.F., Sá L., Kritski A.L. (2004). Defaulting from anti-tuberculosis treatment in a teaching hospital in Rio de Janeiro, Brazil. J. Tuberc. Lung Dis..

[16] Schoeman J.H., Parry C.D., Lombard C.J., Klopper H.J. (1994). Assessment of alcohol screening instruments in tuberculosis patients. Tuberc. Lung Dis..

[17] Rehm J., Parry C. (2009). Alcohol consumption and infectious diseases in South Africa. Lancet.

[18] Muture B.N., Keraka M.N., Kimuu P.K., Kabiru E.W., Ombeka V.O., Oguya F. (2011). Factors associated with default from treatment among tuberculosis patients in Nairobi province, Kenya: A case control study. BMC Public Health.

[19] Finlay A., Lancaster J., Holtz T.H., Weyer K., Miranda A., van der Walt M. (2012). Patient- and provider-level risk factors associated with default from tuberculosis treatment, South Africa, 2002: A case-control study. BMC Public Health.

[20] Dong B., Ge N., Zhou Y. (2001). Smoking and alcohol consumption as risk factors of pulmonary tuberculosis in Chengdu: A matched case-control study. Hua Xi Yi Ke Da Xue Xue Bao.

[21] Kushner M.G., Abrams K., Borchardt C. (2000). The relationship between anxiety disorders and alcohol use disorders: A review of major perspectives and findings. Clin. Psychol. Rev..

[22] Swendsen J.D., Merikangas K.R. (2000). The comorbidity of depression and substance use disorders. Clin. Psychol. Rev..

[23] Graham N.A., Frost-Pineda K., Gold M.S. (2007). Tobacco and psychiatric dual disorders. J. Addic. Disord..

[24] Jané-Llopis E., Matytsina I. (2006). Mental health and alcohol, drugs and tobacco: A review of the comorbidity between mental disorders and the use of alcohol, tobacco and illicit drugs. Drug Alcohol Rev..

[25] Saunders J.B., Aasland O.G., Amundsen A., Grant M. (1993). Alcohol consumption and related problems among primary health care patients: WHO collaborative project on early detection of persons with harmful alcohol consumption—I. Addiction.

[26] Babor T.F., Higgins-Biddle J.C. (2001). Reference. Brief Intervention for Hazardous and Harmful Drinking a Manual for Use in Primary Care.

[27] Freeborn D.K., Polen M.R., Hollis J.F., Senft R.A. (2000). Screening and brief intervention for hazardous drinking in an HMO: Effects on medical care utilization. J. Behav. Health Serv. Res..

[28] Myer L., Smit J., Roux L.L., Parker S., Stein D.J., Seedat S. (2008). Common mental disorders among HIV-infected individuals in South Africa: Prevalence, predictors, and validation of brief psychiatric rating scales. AIDS Patient Care STDS.

[29] Chishinga N., Kinyanda E., Weiss H.A., Patel V., Ayles H., Seedat S. (2011). Validation of brief screening tools for depressive and alcohol use disorders among TB and HIV patients in primary care in Zambia. BMC Psychiatry.

[30] Reid M.C., Fiellin D.A., O’Connor P.G. (1999). Harzardous and harmful alcohol consumption in primary care. Arch. Intern. Med..

[31] Kessler R., Andrews G., Colpe L.J., Hiripi E., Mroczek D.K., Normand S.T., Walters E.E., Zaslavsky A.M. (2002). Short screening scales to monitor population prevalences and trends in nonspecific psychological distress. Psychol. Med..

[32] 32. Kessler R.C., Barker P.R., Colpe L.J., Epstein J.F., Gfroerer J.C., Hiripi E., Howes M.J., Normand S.L., Manderscheid R.W., Walters E.E. (2003). Screening for serious mental illness in the general population. Arch. Gen. Psychiatry.

[33] Brooks R.T., Beard J., Steel Z. (2006). Factor structure and interpretation of the K10. Psychol. Assess..

[34] Andrews G., Slade T. (2001). Interpreting scores on the Kessler Psychological Distress Scale (K10). Aust. N. Z. J. Public Health.

[35] Andersen L.S., Grimsrud A., Myer L., Williams D.R., Stein D.J., Seedat S. (2011). The psychometric properties of the K10 and K6 scales in screening for mood and anxiety disorders in the South African Stress and Health study. Int. J. Methods Psychiatr. Res..

[36] Peltzer K. (2006). Prevalence of alcohol use by rural primary care outpatients in South Africa. Psychol. Rep..

[37] Ward C.L., Mertens J.R., Flisher A.J., Bresick G., Sterling S.A., Little F., Weisner C.M. (2008). Prevalence and correlates of substance use among South African primary care clinic patients. Subst. Use Misuse.

[38] Peltzer K., Davids A., Njuho P. (2011). Alcohol use and problem drinking in South Africa: Findings from a national population-based survey in 2008. Afr. J. Psychiatry.

[39] Parry C.D.H., Rehm J.R., Poznyak V., Room R. (2009). Alcohol and infectious diseases: Are there causal linkages?. Addiction.

[40] Xu L., Gai R., Wang X., Liu Z., Cheng J., Zhou C., Liu J., Zhang H., Li H., Tang W. (2010). Socio-economic factors affecting the success of tuberculosis treatment in six counties of Shandong Province, China. Int. J. Tuberc. Lung Dis..

[41] Pengpid S., Peltzer K., Van der Heever H. (2011). Prevalence of alcohol use and associated factors in urban hospital outpatients in South Africa. Int. J. Environ. Research Public Health.

[42] Johnson T. (2005). Modeling sources of self-report bias in a survey of drug use epidemiology. Ann. Epidemiol..

[43] Allen J.P., Litten R.Z., Fertig J.B., Babor T. (1997). A review of research on the Alcohol Use Disorders Identification Test (AUDIT). Alcoh. Clin. Exp. Res..

[44] Newcombe D.A.L., Humeniuk R., Ali R. (2005). Validation of the world health organization Alcohol, Smoking and Substance Involvement Screening Test (ASSIST): Report of results from the Australian site. Drug Alcoh. Rev..

